# Echocardiographic estimation of left ventricular filling pressures in patients with mitral valve stenosis

**DOI:** 10.5830/CVJA-2013-088

**Published:** 2014-02

**Authors:** Roya Sattarzadeh, Anahita Tavoosi, Parvin Tajik

**Affiliations:** Cardiology Department of Imam Khomeini Hospital, Tehran University of Medical Sciences, Tehran, Iran; Cardiology Department of Imam Khomeini Hospital, Tehran University of Medical Sciences, Tehran, Iran; Department of Clinical Epidemiology, Biostatistics and Bioinformatics, Academic Medical Centre, University of Amsterdam, Amsterdam, the Netherlands

**Keywords:** Doppler echocardiography, tissue Doppler imaging, mitral stenosis, left ventricular end-diastolic pressure

## Abstract

**Background:**

Estimation of left ventricular end-diastolic pressure (LVEDP) among patients with mitral valve disease may help to explain their symptoms. However, conventional Doppler measurements have limitations in predicting LVEDP in this group of patients. The aim of this study was to construct a Doppler-derived LVEDP prediction model based on the combined analysis of transmitral and pulmonary venous flow velocity curves.

**Methods:**

Thirty-three patients with moderate to severe mitral stenosis (MS) who had indications for left heart catheterisation enrolled. Two-dimensional, M-mode, colour Doppler and tissue Doppler imaging indices, such as annular early diastolic velocity (Ea), isovolumic relaxation time (IVRT), pulmonary vein systolic and diastolic flow velocities, velocity propagation, left atrium area (LAA), interval between the onset of mitral E and annular Ea (TE–Ea), and Tei index were obtained. LVEDP was measured in all patients during left cardiac catheterisation. Linear correlation and multiple linear regressions were used for analysis.

**Results:**

The mean of LVEDP was 9.9 ± 5.3 mmHg. In univariate analysis, the only significant relationship was noted with LAA (*p* = 0.05, *R*^2^ = 0.11). However, in multivariate regression, LAA, Tei index and Ea remained in the model to predict LVEDP (*p* = 0.02, *R*^2^ = 0.26). For prediction of LVEDP ≥ 15 mmHg, the best model consisted of LAA, IVRT and Ea, and had a sensitivity of 85% and specificity of 85%.

**Conclusion:**

Our results provided evidence that, in patients with moderate to severe MS, LVEDP can be estimated by combining Doppler echocardiographic variables of mitral flow. However, more studies are required to confirm these results.

## Abstract

Mitral stenosis (MS) is prevalent in developing countries. By improving healthcare systems, it could be expected that the incidence of new cases would decrease and therefore the mean age of mitral stenosis patients would increase. This increase in age of MS patients is accompanied by the occurrence of other diseases, such as coronary artery disease, hypertension, diabetes mellitus and chronic obstructive pulmonary disease.

In a number of patients with MS, the question arises of the impact of mitral valve disease (MVD) on the presenting symptom. For example, in patients presenting with dyspnea, with both significant MS and hypertension, increased left ventricular (LV) filling pressure due to hypertension could influence assessment of the severity of MS. In these patients, severity of MS could be underestimated because the increased diastolic pressure reduces the mitral valve gradient, and the increased LV stiffness shortens pressure half-time (PHT).

Similarly, patients with both pulmonary disease and MS may have dyspnoea because of pulmonary rather than cardiac cause. It is therefore advantageous to assess LV filling pressure in these cases in an attempt to prove or refute a cardiac cause for dyspnoea.

Using Doppler measurements to estimate LV filling pressures is desirable. However, conventional Doppler measurements have limitations in the prediction of left ventricular end-diastolic pressure (LVEDP) in this group of patients. For example, in patients with MS, the left atrium (LA) is enlarged to compensate for the increase in LA pressure. Similarly, mitral inflow peak early diastolic velocity (E) is highly dependent on LA pressure^1^ and also preload.^2^ Pulmonary venous (PV) flow also has a blunted pattern in most patients with MS.^3^ Therefore, in MS patients, LA size, mitral inflow pattern and pulmonary venous pattern are all altered, making these measurements unreliable for the estimation of LVEDP

However, other Doppler and tissue Doppler echocardiographic indices and time intervals, such as peak early diastolic velocity of mitral annulus (Ea), E/Ea ratio, mitral inflow propagation velocity (VP), E/VP, pulmonary vein velocities, Tei index and the ratio of isovolumic relaxation time (IVRT) to interval between the onset of mitral E and annular Ea (TE–Ea), which have shown promising values in the prediction of LV filling pressure in a variety of diseases,^4^-^11^ have not been assessed in the setting of mitral stenosis.

The aim of this study was to analyse the components of mitral and pulmonary waves in patients with mitral stenosis and to construct a Doppler-derived LVEDP prediction model based on the combined analysis of transmitral and pulmonary venous flow velocity curves.

## Methods

The study population comprised 33 consecutive patients with a mean age of 37 ± 9 years, and 23 were women. Inclusion criteria were patients with moderate to severe MS, defined by planimetry as mitral valve area (MVA) of less than 1.5 cm^2^, who were undergoing heart catheterisation, had no more than moderate mitral or aortic regurgitation, and the absence of aortic or tricuspid stenosis. To include these 33 patients, we screened 36 patients. Three were excluded; two had moderate to severe MR and one had moderate to severe AR.

The reasons for undergoing heart catheterisation in patients with MS in our hospital are diagnostic coronary angiography before mitral valve surgery or performing percutaneous transvenous mitral commissurotemy. All patients were evaluated in the left lateral decubitus position by conventional (two-dimensional, M-mode and colour Doppler) and tissue Doppler echocardiography examinations, a maximum of three hours before cardiac catheterisation, by an experienced echocardiologist.

The institutional review board of Imam Khomeini Hospital, which is a tertiary hospital, approved the study protocol. All participants gave written informed consent. This investigation was in accordance with the Declaration of Helsinki.

All Doppler values represent the average of three and 10 beats in sinus and atrial fibrillation (AF) rhythm, respectively. Two-dimensional measurements were performed according to the recommendations of the American Society of Echocardiography.^12^ Mean diastolic transmitral pressure gradient, pressure half-time and mitral valve area by planimetry were calculated. Mitral inflow velocities were measured by pulsed-wave Doppler with the sample volume positioned between the tips of the mitral leaflets in the apical four-chamber view. Peak early diastolic velocity (E), peak late diastolic velocity (A), E/A ratio and deceleration time (DT) were obtained.

**Fig. 1.**
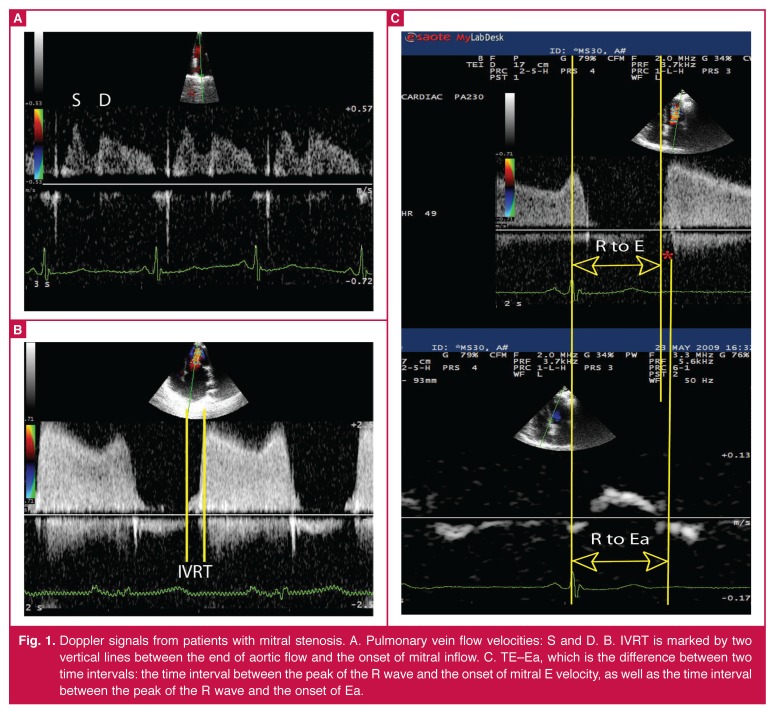
Doppler signals from patients with mitral stenosis. A. Pulmonary vein flow velocities: S and D. B. IVRT is marked by two vertical lines between the end of aortic flow and the onset of mitral inflow. C. TE–Ea, which is the difference between two time intervals: the time interval between the peak of the R wave and the onset of mitral E velocity, as well as the time interval between the peak of the R wave and the onset of Ea.

Mitral inflow propagation velocity was measured as the maximum slope of the first aliasing velocity during early filling from the mitral valve plane to 4 cm distal to the LV cavity in the apical four-chamber view using colour M-mode Doppler. Pulmonary vein systolic flow velocity (PVs) and diastolic flow velocity (PVd) were measured from the apical four-chamber view by placing a sample volume in the right upper pulmonary vein using Doppler echocardiography (Fig. 1A).

Isovolumic contraction time (IVCT), isovolumic relaxation time and ejection time (ET) were assessed by simultaneously measuring the flow into the LV outflow tract and mitral inflow using Doppler echocardiography (Fig. 1B). The index of myocardial performance (IMP or Tei index) was calculated by dividing the sum of IVRT and IVCT by ET.

The pulsed-wave tissue Doppler imaging (TDI) was performed by activating the tissue Doppler function in the same echocardiographic machine. Mitral annulus velocities (myocardial diastolic velocities) were measured using a pulsedwave TDI technique by placing a 1–2-mm sample volume at the level of the septal and lateral annulus. Early diastolic and late diastolic (Aa) velocities of the mitral annulus were determined from the septal and lateral aspects, and the average was calculated.

In addition, ratios such as E/Ea, E/Vp, IVRT/Tei, and PVs/PVs + PVd were calculated. The time intervals between the peak of the R wave and the onset of the mitral E velocity, as well as the time interval between the peak of the R wave and the onset of Ea at the lateral mitral annulus were also measured (Fig. 1C). All Doppler measurements were obtained a maximum of three hours before cardiac catheterisation.

Haemodynamic measurements were done by placing a 6-F fluid-filled catheter in the LV from the right femoral approach under fluoroscopic guidance. The fluid-filled pressure was balanced and calibrated with the external pressure transducer positioned at the mid-axillary level. All recordings were performed before the injection of contrast agent. LV end-diastolic pressure was measured at the nadir of the atrial contraction wave before the onset of rapid LV systolic pressure rise or at the peak of the R wave in a simultaneous ECG if the atrial contraction wave did not exist.

In MS patients undergoing percutaneous commissurotomy, the mean left atrial pressure (LAP) was also recorded. Haemodynamic data were collected at end-expiration by an investigator unaware of the echocardiographic measurements and represented the average of five and 10 cycles in sinus and AF rhythm, respectively.

## Statistical analysis

We described continuous variables as mean ± standard deviation (SD) and categorical data are expressed as frequencies and percentages. Two variables (right atrial area and LV diameter) had > 15% missing data and were omitted from further analysis. Missing values in other variables were imputed using a multiple imputation technique. The first set of imputations was used for further analyses.

Due to the small sample size, we chose to perform a univariate pre-selection of clinically relevant predictors with a *p*-value threshold of 0.3. We then applied a backward selection procedure to develop the final prediction model using linear regression. Model performance was quantified with regard to discrimination [area under the receiver operating curve (AUC)]. The AUC ranges from 0.5 to 1.0 for sensible models. Statistical analyses were done with SPSS for Windows (SPSS Inc, Chicago, Ill), and R for Windows (Version 2.11.1).

## Results

All patients had moderate to severe MS, and 20 (58.8%) had severe MS (MVA ≤ 1 cm^2^). The mean MVA was 0.89 ± 0.19 cm^2^. Less than moderate AI and MR were seen in 60 and 66.7% of patients, respectively. Recording of PV flow was feasible in 30 out of 33 patients (90%). Echocardiographic and haemodynamic characteristics of the patient population are reported in Table 1.

**Table 1:** Summary of haemodynamic and echocardiographic measurements in patients with mitral stenosis

	*Mean ± SD (n = 33)*
Heart rate (bpm)	83.4 ± 20.2
Mean arterial pressure (mmHg)	83.2 ± 10.1
Mean pulmonary pressure (mmHg)	44.3 ± 20.2
LVEF (%)	46.4 ± 7.7
Left atrial area (cm^2^)	28.4 ± 12.2
Average annular Ea (cm/s)	5.5 ± 1.9
Average annular Aa (cm/s)	5.3 ± 1.5
Average E/Ea	38.0 ± 17.5
IVRT (ms)	55.1 ± 10.3
Tei index	0.3 ± 0.1
PVs/PVs + PVd	0.5 ± 0.1
TE–Ea (ms)	23.0 ± 53.0
Velocity propagation (cm/s)	61.0 ± 15.6
E/velocity propagation	0.1 ± 0.01
IVRT/TE–Ea	1.1 ± 4.8

SD, standard deviation; LVEF, left ventricular ejection fraction; Ea, peak early diastolic velocity of mitral annulus; Aa, peak late diastolic velocity of mitral annulus; E, mitral inflow peak early diastolic velocity; IVRT, isovolumic relaxation time; PVs, pulmonary vein systolic flow velocity; PVd, pulmonary vein diastolic flow velocity ; TE–Ea, interval between the onset of mitral E and annular Ea.

The mean LVEDP for the 33 patients was 9.9 ± 5.3 mmHg and ranged from 3–25 mmHg. The results of the univariate analyses are presented in Table 2. In univariate analysis, the only significant relationship was noted with left atrium area (LAA) (*p* = 0.05, *R*^2^ = 0.11). However, in multivariate regression, LAA, Tei index and Ea remained in the model to predict LVEDP (*p* = 0.02, *R*^2^ = 0.26). This model (Table 2) had an area under the ROC curve of 0.71 (95% CI: 0.61–0.80).

**Table 2:** The results of univariate and multivariate linear regression for the prediction of lvedp

*Characteristic*	*Univariate model*			*Multivariate model*	
	*Coefficient (SE)*	*R^2^*	p*-value*	*Coefficient (SE)*	p*-value*
Intercept	–	–	–	–49.51 (6.31)	0.94
LAA	0.38 (0.19)	0.12	0.05	0.43 (0.18)	0.14
Ea	–0.76 (0.50)	0.07	0.14	–0.89 (0.46)	0.02
Tei index	10.95 (8.9)	0.04	0.23	12.30 (8.08)	0.06
E/Ea	10.51 (6.32)	0.08	0.11		
IVRT/TE–Ea	0.33 (0.28)	0.04	0.26		
TE–Ea	0.02 (0.02)	0.03	0.30		
VP	0.06 (0.07)	0.02	0.42		
IVRT	–0.07 (0.1)	0.02	0.46		
E/VP	49.00 (115.34)	0.01	0.67		
PVs/PVs + PVd	–2.73 (13.36)	0.01	0.84		

SE, standard error; LAA, left atrium area; Ea, peak early diastolic velocity of mitral annulus; E, mitral inflow peak early diastolic velocity; IVRT, isovolumic relaxation time; TE-Ea, interval between the onset of mitral E and annular Ea; VP, mitral inflow propagation velocity, PVs, pulmonary vein systolic flow velocity; PVd, pulmonary vein diastolic flow velocity.

We then dichotomised the LVEDP as below 15 and above 15 mmHg. In our series of patients, six had LVEDP ≥ 15 mmHg and the remaining 27 had values below 15 mmHg. The best model for predicting this variable consisted of LAA, IVRT and Ea. The results of univariate and multivariate logistic regression for predicting dichotomised LVEDP (< 15 vs ≥ 15 mmHg) are presented in Table 3.

**Table 3:** The results of univariate and multivariate logistic regression for predicting dichotomised lvedp (< 15 vs ≥15 mmHg)

*Characteristic*	*Univariate model*		*Multivariate model*	
	*Coefficient (SE)*	p*-value*	*Coefficient (SE)*	p*-value*
Intercept	–	–	3.66 (6.25)	0.55
IVRT	–0.09 (0.05)	0.05	–0.16 (0.10)	0.12
LAA	0.20 (0.11)	0.06	0.25 (0.13)	0.06
Ea	–0.39 (0.27)	0.15	–0.62 (0.35)	0.07
Tei index	2.70 (3.88)	0.49		
E/Ea	3.76 (2.60)	0.41		
IVRT/TE–Ea	0.10 (0.14)	0.47		
TE–Ea	0.01 (0.01)	0.41		
VP	0.02 (0.03)	0.50		
E/VP	31.67 (46.45)	0.50		
PVs/PVs + PVd	–4.06 (5.54)	0.46		

SE, standard error; IVRT, isovolumic relaxation time; LAA, left atrium area; Ea, peak early diastolic velocity of mitral annulus; E, mitral inflow peak early diastolic velocity; TE–Ea, interval between the onset of mitral E and annular Ea; VP, mitral inflow propagation velocity, PVs, pulmonary vein systolic flow velocity; PVd, pulmonary vein diastolic flow velocity.

**Fig. 2.**
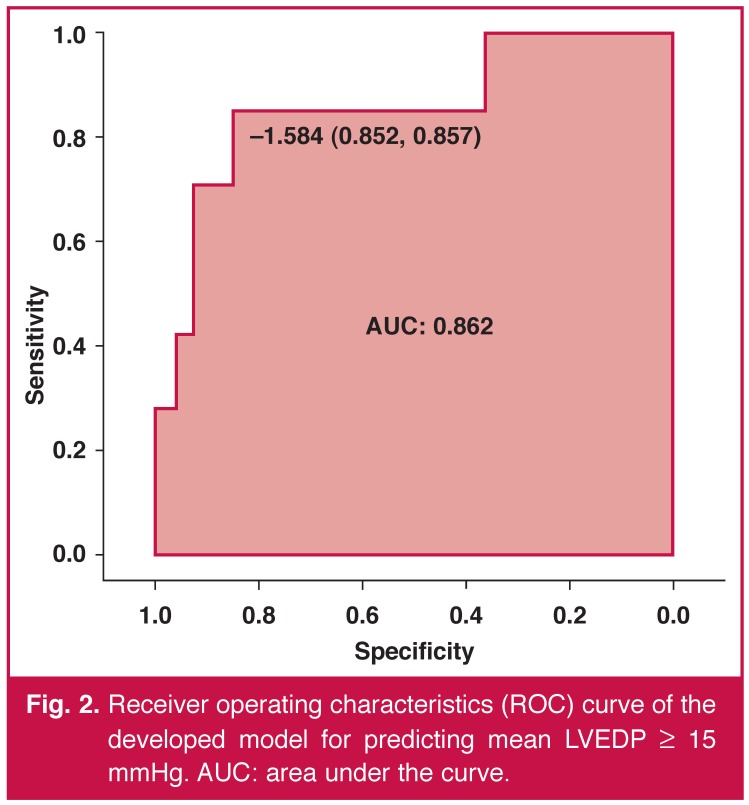
Receiver operating characteristics (ROC) curve of the developed model for predicting mean LVEDP ≥ 15 mmHg. AUC: area under the curve.

For prediction of a mean LVEDP ≥ 15 mmHg and with the use of ROC curves, the model had a sensitivity of 85% and a specificity of 85% Fig. 2. This sensitivity and specificity corresponded to the model value of –1.584. The area under the ROC curve was 0.86 (95% CI: 0.7–1; *p* < 0.001).

The LAP for the 29 patients was 21.6 ± 8.9 mmHg and ranged from 8 to 50 mmHg. In univariate analysis, significant relationships were noted between E/Ea (*p* = 0.005, *r*^2^ = 0.22), E/VP (*p* = 0.032, *r*2 = 0.13), LAA (*p* = 0.013, *r*^2^ = 0.175) and PVs/PVs + PVd (*p* = 0.006, *r*^2^ = 0.21). In multivariate analysis E/Ea, LAA and PVs/PVs + PVd remained in the model to predict LAP (*p* = 0.001, *r*^2^ = 0.39). The results of the univariate and multivariate analyses are presented in Table 4.

**Table 4:** The results of univariate and multivariate analysis for prediction of the mean lap

*Characteristic*	*Univariate model*			*Multivariate model*	
	*Coefficient (SE)*	*R^2^*	p*-value*	*Coefficient (SE)*	p*-value*
Intercept	–	–	–	20.77 (13.92)	0.14
E/Ea	26.40(8.78)	0.22	0.01	17.55 (8.60)	0.05
LAA	0.70 (0.50)	0.17	0.01	0.45 (0.25)	0.08
PVs/PVs + PVd	–52.63 (17.92)	0.21	0.01	–32.57 (17.63)	0.07
Ea	–0.87 (0.76)	0.03	0.25		
IVRT/TE–Ea	0.15 (0.44)	0.01	0.73		
TE–Ea	0.01 (0.03)	0.01	0.55		
VP	–0.02 (0.11)	0.01	0.79		
IVRT	–0.12 (0.15)	0.02	0.42		
E/VP	364.07 (162.38)	0.14	0.03		
Tei index	–4.67 (13.36)	0.01	0.73		

SE, standard error; E, mitral inflow peak early diastolic velocity; Ea, peak early diastolic velocity of mitral annulus; LAA, left atrium area; PVs, pulmonary vein systolic flow velocity; PVd, pulmonary vein diastolic flow velocity; IVRT, isovolumic relaxation time; TE–Ea, interval between the onset of mitral E and annular Ea; VP, mitral inflow propagation velocity.

## Discussion

The present study showed that conventional parameters of LV diastolic function are of limited value in patients with MS. However, it supported a model to estimate LVEDP in patients with significant MS. Interestingly, a number of patients with significant MS had a LVEDP > 15 mmHg, emphasising the importance of assessment of LVEDP in this patient population.

Previous studies have reported on the estimation of mean pulmonary capillary wedge pressure (PCWP) by using mitral inflow in patients with MR,^13^,^14^ and in those with atrial fibrillation.^4^,^15^ In only one study,^16^ patients with MS were included. This study reported weak relationships between PCWP and mitral inflow velocities in patients with MVD, including patients with MS.

In our study, in patients with MS (with and without AF), there were no associations between mitral inflow velocities (E, A, E/A, PHT) and LVEDP or mean LAP. This finding was expected, given the confounding effects of LV relaxation, LV stiffness, LAP and MVA on these measurements.^17^ Patients with MS have a prolonged DT despite an elevated LAP due to valvular stenosis, and DT (or PHT) itself can be used to grade the severity of MS.^1^ It is therefore not surprising that estimation of LV filling pressure from mitral peak diastolic velocities and DT in patients with MVD was inaccurate in our study.

Previous studies have shown that there is a correlation between pulmonary vein parameters and LAP in patients with mitral stenosis. In one study, among the variables of PV flow, the systolic fraction (i.e. the systolic velocity–time integral, expressed as a fraction of the sum of systolic and early diastolic velocity–time integral) correlated significantly with mean LAP (*r* = –0.71, *p* < 0.05) and mitral valve area (*r* = 0.64, *p* < 0.05). Peak velocity and the velocity–time integral in systole also significantly correlated with mean LAP (*r* = –0.66, *r* = –0.67 respectively, *p* < 0.05).^18^

In our study, the relationship of PVs/PVs + PVd with mean LAP reached the level of statistical significance. However, the relationship was weak. There was no relationship between this ratio and LVEDP. It is possible that because PVs/PVs + PVd relates best to mean LAP, we observed no correlations between this ratio and late diastolic LV pressures.

With regard to TDI velocities, our observation was similar to previous studies.^16^,^19^ Ea velocity was reduced in patients with MS, despite a normal EF, and improved the predictive model of LVEDP. It also played a role in discriminate models to predict LVEDP > 15 mmHg.

The accuracy of E/Ea for estimating LV filling pressure appeared to be better in patients with depressed LVEF (< 50%) than in patients with preserved LVEF (≥ 50%).^6^ This ratio (E/Ea) did not improve the prediction of LVEDP in our patients. This may have been because of the presence of a normal LVEF in most of our patients, and confirms an important limitation in using E/Ea in patients with significant MVD.^16^

IVRT has been used for decades in the clinical evaluation of patients with MS, being shorter in patients with more severe MS. LV relaxation also influences IVRT.^16^ All of these make the interpretation of the relationship between IVRT and LVEDP complicated. In our study, although there was no significant correlation between IVRT and LVEDP, this time interval could improve discriminate models to predict LVEDP > 15 mmHg.

Although some previous studies showed strong relationships between the time interval TE–Ea and LV relaxation,^11^ and used this time interval in order to correct for the effect of LV relaxation on IVRT,^16^ we did not observe any relationship between IVRT/TE–Ea ratio and LV filling pressure in patients with MS.

Previous studies have established the value of left atrial size for the prediction of heart failure with both depressed^5^,^20^ and preserved left ventricular systolic function.^21^ In this study the LAA improved the prediction of LV filling pressure in patients with MS and also remained in the discriminate model to estimate LVEDP > 15 mmHg.

There were several limitations to this study. First, there were few patients with MS and LV systolic dysfunction or a depressed EF. Also older patients and those with other cardiovascular diseases (coronary artery disease, hypertension and diabetes mellitus) were absent in our study. There were only six patients with AF in this study. Therefore, we were limited in extrapolating conclusions to these particular subgroups.

Second, in order to obtain meaningful results, a strict and time-consuming methodology must be used, which may limit the everyday application of this method in a busy clinical practice. Therefore the results of our study could be applied in equivocal cases where conventional echocardiography is not matched with the patient’s symptoms.

## Conclusion

Despite these limitations, our results provide evidence that, in patients with mitral stenosis, LV filling pressure can be estimated by combining Doppler echocardiographic variables of mitral flow. However, more studies are required to confirm these results. Doppler echocardiography, a simple, readily available, non-invasive tool, may in future reduce the need for right heart catheterisation in patients with mitral stenosis and unexplained symptoms.
